# Implications of telomeres and telomerase in endometrial pathology

**DOI:** 10.1093/humupd/dmw044

**Published:** 2016-12-15

**Authors:** D.K. Hapangama, A. Kamal, G. Saretzki

**Affiliations:** 1 Department of Women's and Children's Health, Institute of Translational Medicine, University of Liverpool, Liverpool, L8 7SS, UK; 2 Liverpool Women's Hospital NHS Foundation Trust, Crown Street, Liverpool L8 7SS, UK; 3 The National Center for Early Detection of Cancer, Oncology Teaching Hospital, Baghdad Medical City, Baghdad, Iraq; 4 Institute for Ageing and Institute for Cell and Molecular Biosciences, Campus for Ageing and Vitality, Newcastle University, Newcastle upon Tyne, NE4 5PL, UK

**Keywords:** endometrium, telomerase, telomere, stem cells, endometriosis, endometrial cancer, infertility, recurrent miscarriage, progesterone, estrogen

## Abstract

**BACKGROUND:**

Eukaryotic chromosomal ends are linear and are protected by nucleoprotein complexes known as telomeres. The complex structural anatomy and the diverse functions of telomeres as well as the unique reverse transcriptase enzyme, telomerase that maintains telomeres are under intensive scientific scrutiny. Both are involved in many human diseases including cancer, but also in ageing and chronic disease such as diabetes. Their intricate involvement in many cellular processes and pathways is being dynamically deciphered in many organs including the endometrium. This review summarizes our current knowledge on the topic of telomeres and telomerase and their potential role in providing plausible explanations for endometrial aberrations related to common gynaecological pathologies.

**OBJECTIVE AND RATIONALE:**

This review outlines the recent major findings in telomere and telomerase functions in the context of endometrial biology. It highlights the contemporary discoveries in hormonal regulation, normal endometrial regeneration, stem cells and common gynaecological diseases such as endometriosis, infertility, recurrent reproductive failure and endometrial cancer (EC).

**SEARCH METHODS:**

The authors carried out systematic PubMed (Medline) and Ovid searches using the key words: telomerase, telomeres, telomere length, human telomerase reverse transcriptase, telomeric RNA component, with endometrium, hormonal regulation, endometrial stem/progenitor cells, endometrial regeneration, endometriosis, recurrent miscarriage, infertility, endometrial hyperplasia, EC and uterine cancer. Publications used in this review date from 1995 until 31st June 2016.

**OUTCOMES:**

The human endometrium is a unique somatic organ, which displays dynamic telomerase activity (TA) related to the menstrual cycle. Telomerase is implicated in almost all endometrial pathologies and appears to be crucial to endometrial stem cells. In particular, it is vital for normal endometrial regeneration, providing a distinct route to formulate possible curative, non-hormonal therapies to treat chronic endometrial conditions. Furthermore, our current understanding of telomere maintenance in EC is incomplete. Data derived from other malignancies on the role of telomerase in carcinogenesis cannot be extrapolated to EC because unlike in other cancers, TA is already present in proliferating healthy endometrial cells.

**WIDER IMPLICATIONS:**

Since telomerase is pivotal to endometrial regeneration, further studies elucidating the role of telomeres, telomerase, their associated proteins and their regulation in normal endometrial regeneration as well as their role in endometrial pathologies are essential. This approach may allow future development of novel treatment strategies that are not only non-hormonal but also potentially curative.

## Introduction

All eukaryotic chromosomal ends consist of specialized heterochromatin nucleoprotein complexes, termed telomeres, containing repeated nucleotide sequences ((TTAGGG)_n_) and associated specific proteins (Blackburn and Gall, 1978). The intact telomeres prevent the chromosomal ends from being recognized as DNA strand break and protects the loss of genomic DNA as well as end-to-end fusion and degradation of chromosomes. Telomeric DNA is lost with each round of DNA replication (Olovnikov, 1971; Watson, 1971; Lundblad, 2012) and shortening of telomeres beyond a critical length results in a permanent cell cycle arrest. This is due to initiation of sustained DNA damage signalling, resulting in activation of either senescence or apoptosis pathways (Blackburn and Gall, 1978; Blackburn *et al*., 2015). Telomere shortening and telomerase dysfunction are therefore implicated as universal features of cellular senescence and ageing as well as the age-related decrease in tissue regeneration and lifespan restriction in long lived mammals (Djojosubroto *et al*., 2003; Mikhelson and Gamaley, 2012).

The action of the reverse transcriptase enzyme telomerase is the main mechanism that counteracts telomere shortening in cells. Human cells such as embryonic stem cells, germ line cells and cancer cells with unlimited replicative capacity express high levels of telomerase activity (TA) which maintains and elongates telomeres, compensating for telomeric erosion (Counter *et al*., 1992; Yang and Huang, 2014; Meena *et al*., 2015). In contrast, adult stem/progenitor cells (SPCs) have the potential to up-regulate telomerase but these cells also undergo telomere shortening with age (Hiyama and Hiyama, 2007; Flores *et al*., 2008; Rane *et al*., 2016). Most human somatic cells do not express significant levels of TA (Opresko and Shay, 2016) and age-related telomere shortening is commonly described in many human proliferative tissues (Djojosubroto *et al*., 2003) while telomere shortening in post-mitotic tissues is negligible (Benetos *et al*., 2011). Therefore, most work on the functional relevance of telomerase is confined to the aforementioned specialized cells that express telomerase, such as cancer and stem cells.

The human endometrium is a unique organ in terms of regeneration and ageing. It is a dynamic somatic tissue that undergoes repetitive monthly cycles of growth, differentiation, shedding and regeneration throughout a woman's reproductive lifespan. These endometrial cycles of are regulated by ovarian steroid hormones (Hapangama *et al*., 2015). Every month, the endometrium grows from 1 mm in thickness, at the end of the menstrual shedding, to 15 mm in thickness, measured in the mid-secretory phase of the cycle (Dallenbach-Hellweg and Dallenbach, 2010), thus the endometrial regeneration capacity is unparalleled amongst other adult tissues. At the menopause, with the cessation of ovarian steroid hormone synthesis, the endometrium becomes proliferatively quiescent. However, a fully functional endometrium can be regenerated from the remaining thin postmenopausal endometrium with the provision of exogenous ovarian steroid hormones (Paulson *et al*., 2002). Thus, it is the only female reproductive organ not showing irreversible age-related changes. Although it is a somatic organ, endometrium expresses dynamic TA associated with the menstrual cycle. Therefore, the apparent endometrial age-defiance might include a physiological regulation of telomeres and telomerase distinct from other human tissues.

This review focuses particularly on recent findings in endometrial telomere and telomerase biology in the context of the inexhaustible proliferative and regenerative capacity of the human endometrium. This may provide an explanation for the seemingly eternal ‘youthfulness’ retained by the endometrium throughout a woman's life when compared with other female reproductive organs. Furthermore, there is mounting evidence that telomerase and telomere dysfunctions might play important roles in endometrial pathologies.

## Method

We performed systematic PubMed (Medline) and Ovid searches using key words: telomerase, telomeres, telomere length (TL), telomerase reverse transcriptase (TERT), telomeric RNA component (TERC), with endometrium, endometrial SPCs, endometrial regeneration, endometriosis, recurrent miscarriage, infertility, endometrial hyperplasia (EH), endometrial cancer (EC) and uterine cancer. All studies investigating telomerase or telomere biology in endometrium in women or animals or respective cell lines, either primary cells or tissue explants in culture, and published from 1995 until 30th July 2016, were considered. Further manuscripts published before 1995 were also reviewed for specific topic areas and are included as appropriate.

## Telomeres

### Structure

The mammalian telomere complex consists of a tandemly repeated telomeric DNA sequence d(TTAGGG)n and its complementary strand. This is, followed by a short (35–600 nucleotide) single-stranded 3′ guanosine-rich protruding overhang; known as the G-tail (Royale 2006; Blackburn *et al*., 2015). Telomeres are associated with a complex of six well-described shelterin proteins: Telomeric Repeat Factor 1 and 2 (TRF1, TRF2), Repressor/Activator Protein I (RAP1, encoded by TERF2IP gene), TRF1-Interacting Nuclear Protein 2 (TIN2), Tripeptidyl Peptidase I (TPP1) and Protection of Telomeres I (POT1). TRF1 and TRF2 bind directly to the double-stranded telomeric sequence, and POT1 binds the single-stranded overhang; these proteins are therefore telomere DNA binding proteins. They interact with and bind to the remaining three shelterin proteins: TIN2 to TRF1, RAP1 to TRF2 and TPP1 to POT1.

**Figure 1:**
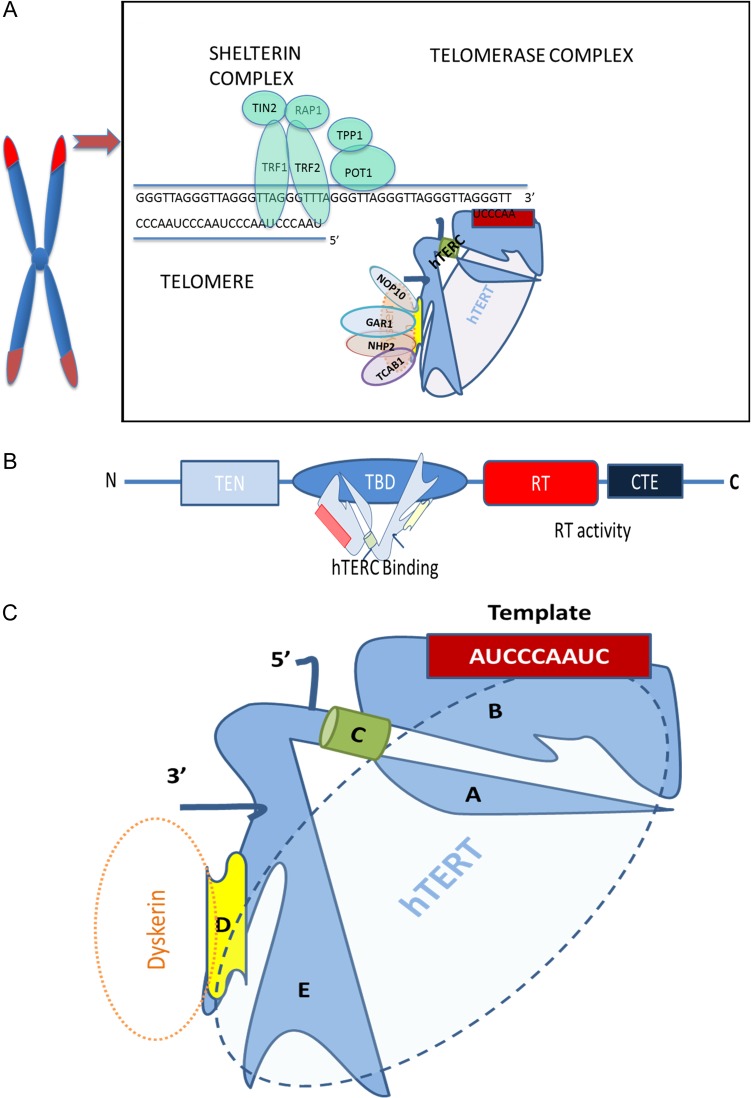
Schematic illustration of the main telomerase subunits and their interaction with the telomere complex. (**A**) Illustration of the human telomere complex and telomerase (only one half of the dimeric holoenzyme complex is shown for clarity). Out of the shelterin proteins, telomere repeat binding factors 1 (TRF1) and 2 (TRF2) bind directly to the double-stranded telomeric sequence, and protection of telomeres protein-1 (POT1) binds to the single-stranded overhang; hence these are named telomere binding proteins and interact with remaining shelterin proteins TIN2 (binds to TRF1), RAP1 (binds to TRF2) and TPP1 (binds to POT1). Telomerase associated proteins NOP10, NHP2 and GAR1 form the H/ACA motif-of the human telomerase reverse transcriptase (hTERT) associated tetramer with dyskerin. (**B**) The four functional domains of hTERT: the telomerase N-terminal (TEN) domain has roles in recruiting telomerase to telomeres as well as telomeric repeat synthesis; telomeric RNA component (TERC)-binding domain (TBD) interacts with hTERC; and both the reverse transcriptase (RT) domain and C-terminal extension (CTE) contribute to the reverse transcriptase enzyme activity (Nakamura *et al*., 1997; Blackburn and Collins, 2011). (**C**) Diagram of the core elements of hTERC: 5′ region containing (A) the pseudoknot domain and (B) stem terminus element-loop that contains the 11 nucleotide RNA template and (C) the template boundary element (Theimer and Feigon, 2006). Both A and B domains are important for *in vivo* stability of hTERC and they interact with hTERT. The RNA stabilizing 3′ region contains (**D**) an H/ACA motif, which interacts with dyskerin or any of the other three H/ACA RNP components (NOP10, NHP2 and GAR1), and (**E**) trans-activating domain containing CR4/5 C that also binds hTERT (Webb and Zakian, 2016). The template boundary element together with the 3′ end prevents DNA synthesis beyond the template (Feng *et al*., 1995; Fu and Collins, 2003; Kiss *et al*., 2010).

The single-stranded overhang forms a D-loop (displacement) (Griffith *et al*., 1999) that prevents the access of telomerase outside of late S-phase when the overhang becomes accessible (Fig. 1A) (de Lange 2005; Palm and de Lange 2008; Shay 2016). In addition, the whole telomere forms a large duplex structure (T-loop) *via* the strand invasion from the 3′ single-stranded overhang (Griffith *et al*., 1999; Blackburn *et al*., 2000) providing proper telomere capping. The T-loop size is believed to be proportional to the length of the respective telomere (Cimino-Reale *et al*., 2001). Thus, in addition to telomere shortening, the dysfunction of telomere capping can also initiate a DNA damage response (DDR) (Griffith *et al*., 1999; Yoo and Chung 2011; Bodvarsdottir *et al*., 2012).

The shelterin complex is ubiquitously expressed and remains associated with telomeres during the cell cycle (Royale 2006; Takai *et al*., 2011). In addition to the six shelterin proteins, there are various additional proteins (e.g. NBS1/MRE11/Rad50, tankyrase, PinX1, Ku) located at the telomere that are involved in DDR and repair processes but also have non-telomeric functions (Kuimov 2004; De Boeck *et al*., 2009).

### Telomere shortening

Telomere shortening with age is a general observation in human proliferating tissues. In cell culture, with each cell division, about 20–50 base pairs (bp) of telomeric DNA is lost due to ‘the end replication problem’ (Olovnikov, 1973; Watson, 1972). This term describes the fact that DNA polymerases can only synthesize in 5′–3′ direction and thus can only synthesize the leading DNA strand un-interrupted. The lagging strand is synthesized by a series of Okazaki fragments which requires the help of short RNA primers and that are finally ligated together by ligases. However, at the very end of the lagging strand, the terminal RNA primer is removed resulting in the 3′ overhang and loss of the DNA in the next round of replication (Watson, 1972; Olovnikov, 1973; Blackburn, 1984; Lundblad, 2012; Mikhelson and Gamaley, 2012; Blackburn *et al*., 2015). In addition to the end replication, environmental conditions such as oxidative stress are an additional mechanism of telomere shortening (von Zglinicki *et al*., 1995; von Zglinicki, 2000). The progressive loss of mean TL is a hallmark of replicative senescence of proliferating cells while the amount of telomere shortening can vary in different tissues and organs during ageing and disease conditions depending on cell proliferation (Benetos *et al*., 2011; Benetos *et al*., 2011) and oxidative stress (von Zglinicki *et al*., 2000). Human mean TLs are 12–15 kb at birth and shorten down to a minimal TL of around 5 kb when a DDR and cell cycle arrest are signalled, which can lead to cellular senescence (Kipling and Cooke, 1990; Calado and Dumitriu, 2013). Shorter telomeres in lymphocytes have been associated with mortality, disease and poor-survival as well as reproductive ageing in humans (von Zglinicki *et al*., 2000; Cawthon *et al*., 2003; Shay, 2016). Thus, TL in human peripheral blood monocytes (PBMCs) has been proposed as a useful biomarker for human ageing and disease (von Zglinicki *et al*., 2000; von Zglinicki, 2002; Bekaert *et al*., 2005).

Although the mean TL of PMBCs had been employed in age determination in forensic medicine, the veracity of this approach is questionable due to the fact that TL is also inherited. In addition, more subtle methods for TL measurements considering initial TL as well as telomere shortening rates have been proposed (Benetos *et al*., 2011; Benetos *et al*., 2013). TL shortening starts during early gestation in many human tissue types such as heart, kidney and brain due to the down-regulation of TA (Ulaner and Giudice, 1997; Ulaner *et al*., 1998, 2001) and fast postnatal organ-specific growth accounts for most of the observed differential organ-specific telomere shortening rates (Carneiro *et al*., 2016).

### Functions of telomeres

#### Prevention of recognition of linear chromosomal ends as double-stranded DNA breaks

The shelterin complex and the telomeric loop structure prevent telomeres from being identified as a DNA break that would signal a DDR. TRFs and POT1 prevent DDR activation due to the formation of the T-loop (Palm and de Lange 2008); TRF2 prevents end-to-end fusion (Cesare and Karlseder 2012) and POT1 helps to prevent the single-stranded telomeric 3′ end from being recognized by the DDR complex by forming a displacement (D-) loop with the remaining double strand (Jacob *et al*., 2007; Yang *et al*., 2008; Baumann and Price, 2010; Kibe *et al*., 2010).

#### Protection of chromosome ends from degradation and end-to-end fusion

Telomeres protect chromosomal ends from degradation by nucleases. Different DNA damage checkpoint proteins act together with EXO1 and MRE11 nucleases to inhibit proliferation of cells undergoing telomere attrition (Keijzers *et al*., 2016; Xue *et al*., 2016). Without the protective capping structure of telomeres, chromosomal ends would fuse together and form anaphase bridges duringmitosis leading to a fuse-breakage-fuse cycle. This process would greatly increase the risk of genomic instability and may result in tumourigenesis (Djojosubroto *et al*., 2003; Meena *et al*., 2015; Shay, 2016; Terali and Yilmazer, 2016).

#### Sentinels for DNA damage

Telomeres are more susceptible to DNA damage than genomic DNA (Petersen *et al*., 1998) due to their high guanine content (Henle *et al*., 1999; Wang *et al*., 2010) and lack of DNA repair mechanisms (Petersen *et al*., 1998). Telomere-associated DNA damage in the form of TIFs (telomere dysfunction-induced foci) or TAFs (telomere-associated foci) is hardly ever repaired (d'Adda di Fagagna *et al*., 2003; Takai *et al*., 2003; Takai *et al*., 2011; Fumagalli *et al*., 2012; Hewitt *et al*., 2012). This telomere-associated damage can have the same function as critically shortened telomeres in signalling cell cycle arrest. As the ‘first responders’ to hazards of genomic instability, the damaged telomeric DNA initiates a sustained DDR, resulting in a cell cycle arrest and inducing senescence or apoptosis, thereby protecting the organism from dangerous genetic aberrations and mutations (Blackburn *et al*., 2015; Shay 2016). Telomeres have thus been proposed to be sentinels for DNA damage (von Zglinicki 2002) and epigenetic sensors of general stress in DNA metabolism (Cesare and Karlseder 2012).

#### Recruitment of telomerase and regulation of its access to telomeres

Shelterin (Fig. 1) has a dual role in recruitment of telomerase and blocking its access to telomeres (Smogorzewska and de Lange 2004; Palm and de Lange 2008; Nandakumar and Cech 2013; Zhang *et al*., 2013; Schmidt and Cech 2015). POT1 prevents telomerase accessing an intact telomere complex but after hetero-dimerization with TPP1, it allows telomerase to become active at telomeres and to extend the 3′ overhang in late S-phase (Wang *et al*., 2007; Zhang *et al*., 2013; Chu *et al*., 2016).

#### Regulation of gene transcription/telomere position effect

Telomeres may also regulate gene transcription *via* a telomere position effect (TPE) (Robin *et al*., 2014), whereby genes located close to the telomeres are transcribed at a reduced rate. This allows changeable epigenetic transcriptional repression permitting genes the ability to switch their transcription rate. TPE has been reported to affect the expression of genes involved in stress, growth and recognition by the immune system in various invertebrate organisms and in cultured human cells (Robin *et al*., 2014). Recently it has also been connected to human diseases (Stadler *et al*., 2013). Further work examining the role of TPE in gene regulation in human tissues and during telomere shortening is needed to unravel its involvement in endometrial diseases.

#### Non-telomeric functions of telomere-associated proteins

Some shelterin proteins also have non-telomeric, genomic binding sites that allow extra-telomeric functions, such as regulating transcription of various genes. For example, RAP1 has been shown to regulate female obesity, a function unrelated to telomeres (Martinez *et al*., 2013). Cell type, subcellular localization and development stage specific pathways may regulate the shelterin complex. TIN2 has been found in mitochondria (Chen *et al*., 2012) and a reduction in TIN2 expression inhibits glycolysis and reactive oxygen species production and enhances ATP levels and oxygen consumption in cancer cells. This suggests a link between some shelterin proteins and metabolic control, providing an additional mechanism by which telomeric proteins might regulate the cellular processes beyond their function at telomeres. Additional non-telomeric functions in embryonic development have also been described for TIN2 (Chiang *et al*., 2004).

### Telomere maintenance

Cells can maintain their telomeres *via* a telomerase dependent pathway or a telomerase independent alternative lengthening of telomeres (ALT) pathway (Brien *et al*., 1997; Bryan *et al*., 1997). Although activation of the latter pathway has been limited to particular types of cancers (sarcomas) and immortalized cell lines, there are suggestions that the ALT process may occur under physiological conditions in undifferentiated cells such as stem cells or even normal somatic cells (Neumann *et al*., 2013; Bojovic *et al*., 2015). There is a general consensus that in telomerase competent cells and in most normal cells, the ALT process is redundant and hence repressed (Henson *et al*., 2002). Therefore, in the context of the endometrium, ALT is less likely to be relevant and this review focuses mainly on telomerase dependent telomere maintenance.

## Telomerase

Telomerase is a reverse transcriptase (RNA dependent DNA polymerase) that employs an integral RNA subunit harbouring a template sequence to add G-rich telomeric repeats to the 3′ single-stranded overhang of telomeres (Lingner *et al*., 1997). The telomerase holoenzyme has a dimeric structural configuration, where each half contains a human TERT (hTERT) and human TERC (hTERC) connected by a hinge region in the middle (Blackburn, 2011; Blackburn and Collins, 2011) (Figs 1B and C). The main components of the telomerase complex are hTERT, hTERC and dyskerin (DKC1) (Venteicher *et al*., 2009) (Fig. 1C). In addition, there are various telomerase associated proteins that interact with these core components (Fig. 1A). There are 184 telomeres and approximately 250 molecules of telomerase and in a cancer cell in late S-phase, when telomerase is actively recruited to telomere ends (Mozdy and Cech, 2006; Xi and Cech, 2014; Schmidt and Cech, 2015). When not active at telomeres, telomerase is localized to Cajal bodies in the nucleus for most of the cell cycle. After the telomerase/telomere interaction, every single telomerase activation event is thought to add 50–60 nucleotides to most telomeres in cancer cells with short TLs *in vitro* (Zhao *et al*., 2009; Schmidt and Cech 2015). Since the telomere lengthening action is limited to the nucleus, shuttling of the telomerase protein hTERT out of the nucleus prevents any telomeric extension. This shuttling is regulated by different domains on the hTERT protein; for example, a nuclear localization signal at amino acid residues 222–240 of hTERT (Chung *et al*., 2012) and a nuclear export signal (Seimiya *et al*., 2000) as well as a mitochondrial localization signal (Santos *et al*., 2004) have been described. Furthermore, recent data also suggest that the ability of telomerase in extending telomeres may be dependent on pH levels (Ge *et al*., 2016). Acidic pH (6.8) encourages preferential lengthening of short telomeres yet telomerase lengthens telomeres independent of their lengths at higher pH levels (7.2, 7.4).

### Telomerase components

Telomerase reverse transcription activity has been demonstrated in an *in vitro* cell free system with just hTERT and hTERC (Weinrich *et al*., 1997). However, some compounds such as dyskerin actively associate with the telomerase complex in a cellular environment and are important for stability, maturation and function of the enzyme (Cohen *et al*., 2007).

#### Human telomerase reverse transcriptase

hTERT is the catalytic subunit of telomerase and is often the main rate-limiting factor for telomerase enzyme activity (Zhou *et al*., 2006; Zhang *et al*., 2013). The hTERT gene is located on chromosome 5p15.33 (Shay 2010) and consists of 16 exons and 15 introns spanning ∼35 kb (Cong *et al*., 1999). There are over 20 spliced variants of hTERT but only the wild type (full lengths protein, Fig. 1B) exhibits reverse transcriptase activity (Hrdlickova *et al*., 2012). The balance between the full lengths hTERT and its different splice variants has been shown to affect its function (Listerman *et al*., 2014; Radan *et al*., 2014). In addition to telomere maintenance, hTERT is implicated in increasing the anti-apoptotic capacity of cells, maintaining pluripotency of stem cells and regulating gene expression (for review see Saretzki, 2014).

#### Human telomeric RNA component

TERCs are species specific in size and sequence, but highly conserved in their structure and all contain a short sequence complementary to the telomeric TTAGGG hexanucleotide repeat sequence. Human TERC is relatively short (451 nucleotides (nt) compared with >1000 nt in yeast) (Theimer and Feigon 2006). The 3′ stabilizing element shares an H/ACA motif with small nucleolar and small Cajal body RNAs (snoRNA, scaRNA), and in turn associates with all four H/ACA RNP components, dyskerin, NOP10, NHP2 and GAR1 (Egan and Collins 2010) (Fig. 1A). hTERC is a non-coding RNA transcribed by RNA polymerase II (Gallardo and Chartrand 2008; Smekalova *et al*., 2012). It undergoes subsequent exonucleolytic cleavage up to the boundary formed by the H/ACA domain, meaning its co-transcriptional association with dyskerin is essential for stabilization, preventing further cleavage and nuclear retention (Feng *et al*., 1995; Fu and Collins 2003; Kiss, Fayet-Lebaron and Jady 2010). This H/ACA domain is mutated in dyskeratosis congenita, where the disease-associated hTERC variants impair hTERC accumulation. Disease-associated hTERC variants with sequence changes outside the H/ACA domain do not affect hTERC RNA processing or stability; they instead impose a catalytic defect (Fu and Collins 2003). The tetrameric complex of the accessory proteins dyskerin, NOP10, NHP2 and chaperone NAF1 (which later is replaced by GAR1) bind to hTERC and this association is crucial for normal TA.

#### Dyskerin

Dyskerin is an evolutionarily conserved 58 kDa, 514-amino-acid large protein (Knight *et al*., 1999). In humans, it is encoded by the DKC1 gene located on chromosome Xq28 (Cerrudo *et al*., 2015) and it is generally located in the nucleus. Dyskerin is an essential protein for cellular survival; thus DKC1 deletion is lethal (Rocchi *et al*., 2013; Angrisani *et al*., 2014). In the context of telomerase, dyskerin plays an established role in the maintenance of telomere integrity by stabilizing hTERC in the telomerase holoenzyme that is assembled in Cajal bodies (Cohen *et al*., 2007; Gallardo and Chartrand 2008; Gardano *et al*., 2012). Dyskerin is the only component to co-purify with active, endogenous human telomerase (Gardano *et al*., 2012). Loss of dyskerin binding leads to hTERC degradation and reduction in TA *in vivo* (Shukla *et al*., 2016). Furthermore, dyskerin has other non-telomerase associated functions essential to elementary cellular events such as mRNA translation, growth and proliferation. Dyskerin may regulate these functions *via* directing the isomerization of specific uridines to pseudouridines by acting as a catalytic pseudouridine synthase and by acting through the snoRNA-derived miRNA regulatory pathway, thus affecting different biological processes (reviewed in Angrisani *et al*., 2014).

#### Other accessory proteins of telomerase

Apart from NOP10, NHP2 and GAR1 which form the H/ACA motif-associated tetramer with dyskerin (Fig. 1), there are a plethora of other proteins (some listed in Table I) associated with telomerase with roles including: assembly, processing of telomerase, localization and accessibility to telomeres. In addition to these proteins, telomerase interacts with many others which are required for the formation of the appropriate structure and its stabilization, however their importance in TA is unknown (Smekalova *et al*., 2012). It is important also to appreciate the close relationship of telomerase with many cell cycle regulating, tumour suppressor, pluripotency and epithelial–mesenchymal transition (EMT) related proteins and pathways, such as Wnt/β-catenin, Cyclin D1, BCL-2, OCT-4, p53, EGFR, etc. (Ding *et al*., 2011; Listerman *et al*., 2014; Tang *et al*., 2016; Xue *et al*., 2016). Interestingly, recent data has suggested that the DDR kinases Ataxia Telangiectasia Mutated (ATM) and Ataxia Telangiectasia and Rad3-Related Protein (ATR) are required to recruit telomerase to telomeres *via* a TRF1 regulated pathway (Tong *et al*., 2015), while a central role of the ATM pathway in regulating telomere addition has been further highlighted (Lee, Bohrson, *et al*., 2015). In yeast, there exists a counting mechanism involving the shelterin RAP1, which prevents ATM accessing/activating telomerase on long telomeres thereby regulating TL (Yuan *et al*., 2013). However, whether a similar feedback mechanism exists in human cells is not yet known (Runge and Lustig, 2016). These findings are only beginning to unravel the intricate cellular pathways that are converging to regulate telomerase and telomere biology.

**Table I:** Telomerase associated proteins.

hTERT associated proteins	Function
P23, hsp90	Assist in ribonucleoprotein (RNP) assembly (Elmore *et al*., 2008).
Protein 14-3-3	Regulates signal transduction and apoptosis Prevents nuclear export of hTERT Regulates localization of the hTERT binding partners (Xi and Cech, 2014)
DHX36 (DEAH-Box Helicase 36) Protein	Stabilizes hTERT Corrects positioning of the template domain of hTERT (Sexton and Collins, 2011)
Nuclear VCP-Like protein 2 (NVL2)	Knockdown reduces telomerase activity (Her and Chung, 2012)
Pontin and reptin	Facilitate assembly of the minimally active enzyme consisting of TERT, TERC and dyskerin (Huber *et al*., 2008)
**hTERC associated proteins**	**Function**
GAR1, NAP57, NOP10, NHP2 (members of H/ACA ribonucleoproteins complex)	Associated with stability, accumulation, maturation and localization of hTERC Regulate cell proliferation (Wang and Meier, 2004)
Dyskerin (DKC1, a member of H/ACA ribonucleoproteins complex)	Stabilizes TERC Reduces genetic instability Effective RNA pseudouridylation loss of dyskerin arrests the cell cycle (Ashbridge *et al*., 2009; Egan and Collins, 2010)
A1, UP1	Help in accessibility of telomerase to telomeres (Fiset and Chabot, 2001; Nagata *et al*., 2008)
TEP1 (telomerase protein component 1)	Not essential for telomerase activity or telomere length maintenance *in vivo*, essential for telomere replication (Harrington *et al*., 1997)
La antigen	Direct and specific interaction between La and TERC influences telomere homeostasis (Aigner *et al*., 2003)
STAU (human homologue of staufen), RPL22 (Ribosomal Protein L22)	Assist in telomerase processing, localization and telomerase assembly (Le, Sternglanz and Greider, 2000)
TCAB1 (telomerase and Cajal body protein 1, encoded by WRAP53)	May license the catalytically active hTERT–hTERC holoenzyme for recruitment to telomeres (Venteicher *et al*., 2009)

### Functions of telomerase

#### Telomere maintenance

In eukaryotic cells, TA counteracts the end-replication problem and elongates the 3′ single strand in the absence of a DNA template. The subsequent replication of the complementary C-rich strand then will be possible by the conventional DNA replication in the next S-phase. The human telomerase complex consisting of hTERC and hTERT is targeted to telomeres specifically in the late S-phase of the cell cycle (Hug and Lingner 2006). Recent work has suggested that hTERT remains bound to hTERC for most of the cell cycle (Vogan and Collins 2015). The telomerase holoenzyme Cajal body-associated protein, TCAB1, is released from hTERC during cell cycle progression in mitotic cells coincident with TCAB1 delocalization from Cajal bodies (Vogan and Collins 2015). This observation proposes that TCAB1 and hTERC association may license the catalytically active hTERT–hTERC holoenzyme for recruitment to telomeres in the G1 phase of the cell cycle. TRF1 is the shelterin protein that is primarily responsible for regulation of an efficient replication of telomeric DNA (Sfeir *et al*., 2009). Apparently not all telomeres are required to be elongated by telomerase in each DNA replication round but there might be preferential lengthening of the shortest telomeres in telomerase active cells to ensure all the TLs remain above a critical length that would otherwise initiate activation of apoptotic and cell cycle arresting pathways (Fakhoury *et al*., 2010; D'Souza *et al*., 2013). The exact mechanism by which telomerase differentially extends telomeres with various lengths is still not well understood.

#### Non-telomeric functions of the telomerase component hTERT

Although telomere protection/lengthening is the most widely studied function of telomerase, there has been a considerable amount of evidence on non-telomeric functions of the protein subunit hTERT, such as promoting cellular proliferation/growth, survival, retaining an undifferentiated status, as well as increasing motility and metabolism (reviewed in Saretzki, 2014; Terali and Yilmazer, 2016). Recent evidence also suggests that hTERT binds to and stimulates ribosomal DNA transcription, particularly under hyper-proliferative conditions (Gonzalez *et al*., 2014). Protection of mitochondrial function under oxidative stress has been proposed as an important role of hTERT in various cell types, it was associated with reduction in oxidative stress and sensitivity to apoptosis as well as with a reduction in DNA damage (Ahmed *et al*., 2008; Singhapol *et al*., 2013). While initial studies on the beneficial role of mitochondrial hTERT were conducted mainly *in vitro* in cell culture, recent data describe beneficial effects also *in vivo*, for example, in vascular function (Beyer *et al*., 2016). Telomerase may interact with various well-established proliferative pathways including EGFR signalling (Smith *et al*., 2003), MYC and Wnt/β-catenin pathways (Park *et al*., 2009; Hoffmeyer *et al*., 2012). Telomerase has been shown to promote cell survival by blocking the death receptor (Dudognon *et al*., 2004) as well as by down regulating pro-apoptotic genes such as BAX and BCL-2 (Del Bufalo *et al*., 2005; Massard *et al*., 2006) in addition to suppressing mitochondrial and endoplasmic reticulum stress induced cell death (Zhou *et al*., 2006). Telomerase inhibition in stem cells has induced differentiation and loss of pluripotency genes, suggesting a role as a pluripotency gene in embryonic stem cells maintaining an undifferentiated status (Saretzki *et al*., 2008; Yang *et al*., 2008; Liu *et al*., 2013).

## The endometrium

**Figure 2:**
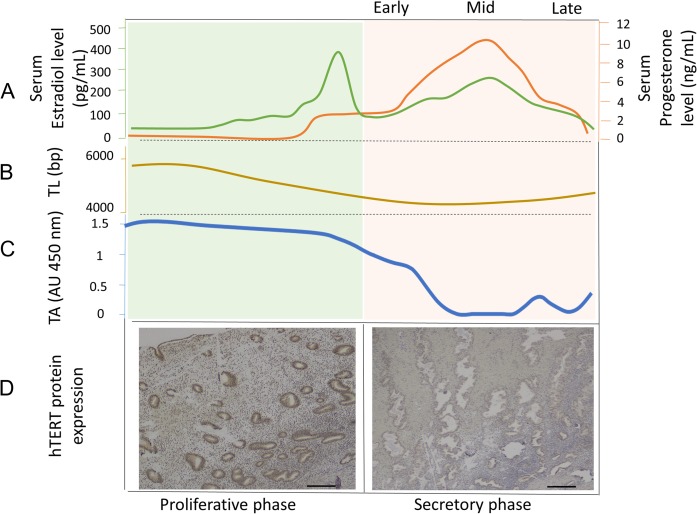
Correlation of typical ovarian hormonal changes with the observed changes in endometrial telomerase activity (TA), mean telomere length (TL) and endometrial hTERT protein expression. (**A**) Estrogen (green line) and progesterone (orange line) (ovarian hormones) show typical cyclical variations during the menstrual cycle in premenopausal women. (**B**) Endometrial TA increases steadily under the influence of estrogen in the proliferative phase, whereas the levels plummet in the progesterone-dominant secretory phase of the cycle (Kyo *et al*., 1997; Saito *et al*., 1997; Williams *et al*., 2001; Hapangama, Turner, Drury, Quenby, *et al*., 2008; Hapangama *et al*., 2009). (**C**) Our recent work further demonstrates similar dynamic changes in the mean endometrial TL across the menstrual cycle (Valentijn *et al*., 2015). (**D**) In full thickness endometrial tissue sections, hTERT protein expression studied with immunohistochemistry employing a monoclonal mouse anti-human telomerase antibody (ab27573, Abcam, Cambridge UK), detection with ImmPRESS anti-mouse/rabbit polymer and visualization with ImmPACT DAB (Vector Laboratories, Peterborough, UK). Positive hTERT staining was observed in functionalis and basalis epithelial cells in the proliferative phase but the brown positive staining is limited to the basalis epithelium in the secretory phase. Magnification ×200, Scale bar 10 μm.

Human endometrium lines the uterine cavity and is organized into two functionally distinct layers: the superficial functionalis and deeper basalis (Valentijn *et al*., 2013); (Hapangama, Kamal and Bulmer 2015). The transient, exquisitely hormone responsive, functionalis exists only during the reproductive life of a woman, whereas the permanent, relatively hormonally unresponsive basalis layer persists throughout her whole life. The endometrial menstrual cycle is an exclusive phenomenon to upper order primates and is regulated by ovarian steroid hormonal signals (Hapangama *et al*., 2015; Kamal *et al*., 2016; Kamal *et al*., 2016) (Fig. 2A).

Endometrium is the primary target organ for ovarian steroid hormones and the endometrial cell cycle is intricately regulated by them. The reproductive lifespan of a woman is dictated by ovarian function. It commences with menarche and finishes with menopause. During that period, an average woman endures about 400 menstrual cycles in which the functionalis layer of the endometrium undergoes a well-defined cycle of proliferation, differentiation and menstrual shedding followed by regeneration. Ovarian steroid hormones regulate this endometrial cycle *via* their cognate receptors (reviewed in Hapangama *et al*., 2015; Kamal *et al*., 2016). It is generally accepted that estrogen is the trophic hormone of the endometrium, where it induces cellular growth and proliferation; while progesterone influences cellular differentiation and counteracts proliferation and other estrogenic effects (Hapangama, 2003). The third ovarian steroid hormone, androgen, is also postulated to impact the endometrial cycle, yet unlike the aforementioned hormones, the exact details of androgenic regulation of the endometrium are yet to be fully elucidated. The huge regenerative potential seen with the monthly endometrial cycle is unparalleled by other tissues, and the exact reason for this menstrual shedding, which is biologically a very expensive process, is yet unknown.

### Telomerase and telomeres in endometrial tissue: functional relevance

TA is high in the premenopausal endometrial functionalis (Kyo *et al*., 1997) (Fig. 2C and D). Its dynamic changes regulated by the ovarian cycle are well established and correlate with glandular proliferation (Williams *et al*., 2001; Hapangama *et al*., 2008; Hapangama *et al*., 2009). Our recent work further demonstrates similar dynamic changes in the mean endometrial TLs across the menstrual cycle (Valentijn *et al*., 2015) (Fig. 2B). Once the ovarian hormone production has ceased, the relatively quiescent postmenopausal endometrium expresses low levels of TA (Brien *et al*., 1997; Tanaka *et al*., 1998).

**Figure 3:**
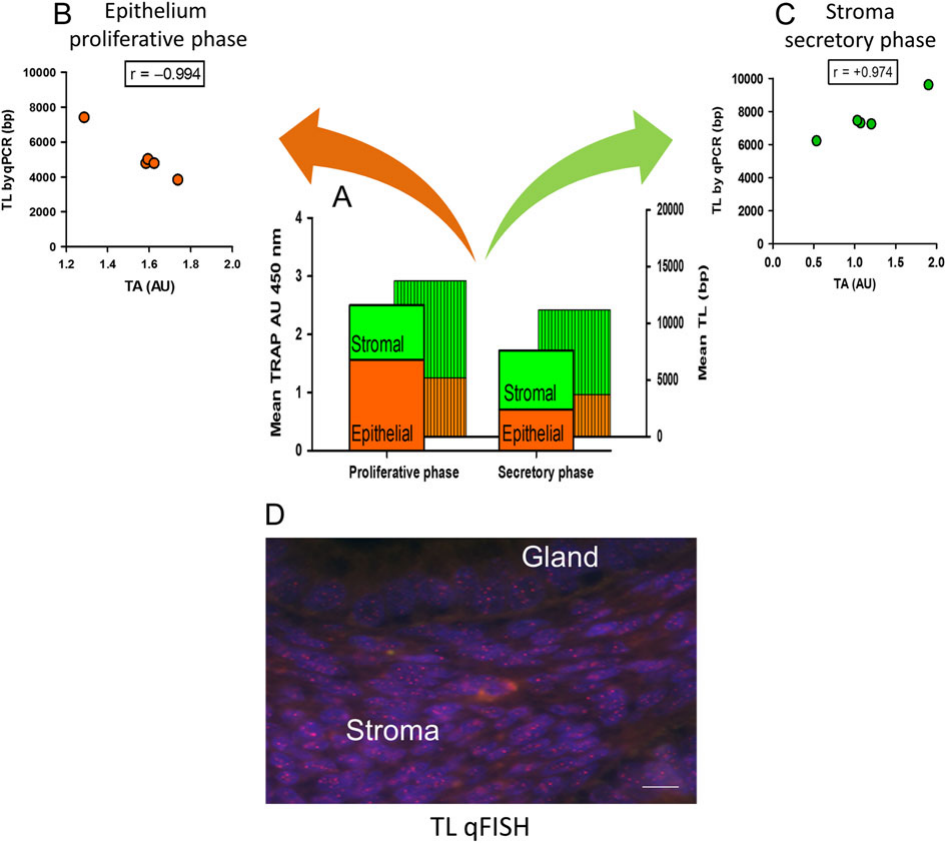
TLs and TA in the human endometrium. (**A**) Endometrial TA (measured by TRAP assay) with endometrial TL (measured by qPCR) during the proliferative and the secretory phase of the cycle in healthy women with proven fertility (Valentijn *et al*., 2015). (**B**) TA correlated negatively with TL in isolated epithelial cells in the proliferative phase (*n* = 5, *r* = −0.994, ***P = 0.0005). (**C**) TA correlated positively with TL in endometrial stromal cells in the secretory phase (*n* = 5, *r* = +0.974, ***P = 0.0005); no correlation was seen between these parameters during the proliferative phase in the stroma or the secretory phase of the epithelium. Epithelia represent Epcam +ve epithelial fraction (positive selection) and stroma represents Epcam −ve stromal cell fraction from the dissociated endometrial biopsies. Single cell suspensions were purified using Epcam microbeads (negative selection) (Valentijn *et al*., 2015). (**D**) Telomeres identified in an endometrial sample during the proliferative phase by fluorescence *in situ* hybridization (FISH) using a peptide nucleic acid telomere probe (Panagene, Japan). Note the brighter (red) telomere signal in the stromal cells compared to the epithelial cells. Scale bar 50 µM.

When examining distinct cellular compartments within the endometrium, stromal cells, regardless of the cycle phase, maintain longer TLs compared with the epithelial cells. However, they demonstrate absent or significantly lower TA (Fig. 3A–C) and hTERC expression compared with the epithelial cells (Tanaka *et al*., 1998; Yokoyama *et al*., 1998; Valentijn *et al*., 2015), (Vidal *et al*., 2002). Data from a previous study which employed *in situ* assessment of endometrial TLs also demonstrated that glandular epithelium of the endometrial functionalis possesses the shortest TL (Cervello *et al*., 2012) (Fig. 3D). Furthermore, the proliferating endometrial epithelial cells have the highest TA that correlates negatively with TL (Valentijn *et al*., 2015) (Fig. 3B). This suggests that in the epithelial cells, the high TA preferentially preserves the short telomeres in order to avoid a critically short length. In rodents, estrogen increases the pH in the uterine fluid, while progesterone has the opposite effect (Chinigarzadeh *et al*., 2016). It can therefore be speculated that low pH in the proliferative phase may preferentially direct telomerase function to the short telomeres in endometrial epithelial cells. This is in accordance with the recent evidence regarding pH dependent telomerase function (Ge *et al*., 2016). Further studies would be required to fully investigate and confirm this possibility.

Moreover, endometrial hTERT may have extra-telomeric functions. Direct *in vitro* inhibition of TA with the TERC inhibitor ‘imetelstat’ inhibited endometrial cell proliferation and disrupted gland formation by healthy epithelial cells (Valentijn *et al*., 2015). In contrast, overexpressing hTERT in endometrial stromal cells did not increase cell proliferation rate or hormone responsiveness (Barbier *et al*., 2005) similar to the other non-endometrial fibroblasts (Ahmed *et al*., 2008). Thus, there might be a specific co-regulation of TA and proliferative capacity limited to the endometrial epithelial cells or to epithelial cells in general. Other groups have found a correlation between TA in ovarian granulosa cells and their proliferation and differentiation status which is also under the control of growth factors and steroid hormones, similar to endometrial epithelium (Chronowska 2012). Importantly, although it has been possible to immortalize benign endometrial stromal cells by overexpressing hTERT (Krikun *et al*., 2006), immortalization of endometrial epithelial cells using a similar process has not been equally successful. This might be due to the fact that epithelial cells are likely to require an additional inhibition of the p16INK4a tumour suppressor in order to be immortalized by hTERT overexpression (Kiyono *et al*., 1998; Farwell *et al*., 2000; Shao *et al*., 2008; Novak *et al*., 2009).

The previously reported immortalization of benign human endometrial epithelial cells with hTERT overexpression (Kyo *et al*., 2003) has not been successfully replicated. This is an important fact, as the only other supposedly immortalized benign endometrial epithelial cell line that was generated by telomerase overexpression (Hombach-Klonisch *et al*., 2005) was later confirmed to be the misidentified breast cell line MCF-7 (Korch *et al*., 2012). This particular cell line was widely available to several groups leading to many publications (King *et al*., 2010). Unfortunately, the reportedly immortalized epithelial cell line generated by Kyo and colleagues (Kyo *et al*., 2003) has not undergone similar scrutiny and has not been available to other groups for further confirmatory studies for authenticity. In summary, we conclude that there are fundamental differences in telomerase function between endometrial epithelial and stromal cells. The mere presence of telomerase may result in a survival advantage for stroma, while epithelial cell proliferation may be regulated by telomerase. Additional factors than TA seem to be necessary for the long term survival and immortalization of epithelial cells in culture.

We are just beginning to understand the importance of extra-telomeric functions of telomere and telomerase components; for example, RAP1 has an extra-telomeric function on stromal cell decidualization in the rat endometrium (Kusama *et al*., 2014). Further examination of extra-telomeric functions of telomerase/telomeric proteins in the endometrium is warranted to further reveal their interplay with other cell cycle regulators specific to the endometrium.

### Hormonal regulation of telomerase in epithelial cells

TA can be regulated at multiple levels, e.g. transcription, splicing, epigenetic and post-translational modification (reviewed in Fojtova and Fajkus, 2014; Lewis and Tollefsbol, 2016; Akincilar *et al*., 2016). Most human somatic tissue has absent or low levels of TA which is tightly regulated compared with the high and easily detectable levels seen in cancer cells and in germ line/stem cells. Thus, the initial studies on telomerase regulation were conducted in the context of developmental or stem cell biology or in cancer cells (reviewed in Batista, 2014; Huang *et al*., 2014; Gladych *et al*., 2011). However, in this review, we focus primarily on the hormonal regulation of telomerase at a normal, physiological level in the endometrium which is pivotal to its function.

#### Estrogen

Early work on the hormone responsive breast cancer cell line MCF-7 showed that estrogen up-regulates TA and hTERT gene expression *via* direct and indirect effects on the hTERT promoter (Kyo *et al*., 1999). Gel shift assays on MCF-7 cells further revealed that there is an imperfect palindromic estrogen-responsive element in the hTERT promoter that specifically binds to estrogen receptor (ER) and is responsible for transcriptional activation by ligand-activated ER (Kyo *et al*., 1999). Further confirmation of 17β-estradiol (E2) induced transcription of hTERT *via* ERα was also reported in various other cell types, including ovarian epithelial cells (Kimura *et al*., 2004; Gladych *et al*., 2011), ovarian stromal cells (Misiti *et al*., 2000), mesenchymal stem cells (MSCs) (Cha *et al*., 2008) and human umbilical vein endothelial cells (Hiyama and Hiyama 2007). Although ChIP assays in prostate cells suggested a recruitment of both ER subtypes to the hTERT promotor, its induction by ERβ in other cells remains controversial (Nanni *et al*., 2002).

There is some evidence that longer exposure to endogenous estrogen (length of reproductive years of life) might correlate with greater TLs and TA in PBMCs (Pines 2013). In other words, longer TLs seem to be present in different tissues and may be associated with longer reproductive life. E2 increased TA and TERT mRNA in heart, liver and brain tissue in an ovariectomized rat model (Cen *et al*., 2015). However, mature peripheral T cells do not respond to E2 with changes in expression or function of telomerase (Benko *et al*., 2012) suggesting that the effect of estrogen on telomerase is tissue/cell specific. Finally, the relative longevity of women compared with men has been speculated to be related to the effects of estrogen induced telomerase on telomere protection (Leri *et al*., 2000; Calado *et al*., 2009; Barrett and Richardson 2011; Gopalakrishnan *et al*., 2013; Cen *et al*., 2015). More active telomerase was found in cardiac myocytes from female rats which seems to correspond to higher myocyte numbers in older women compared to myocyte loss in older men (Leri *et al*., 2000). Others have suggested that reduction of oxidative stress by estrogens may result in longer telomeres in tissues such as brain and liver (Vina *et al*., 2005). Greater female longevity is suggested to possibly be connected to the female exposure to estrogens (Muezzinler *et al*., 2013). However, a recent longitudinal study reports a higher rate of PBMC TL attrition in the premenopausal period than in the postmenopausal period (Dalgard *et al*., 2015) with the authors proposing the opposite effect of estrogen on leucocyte turnover and menstrual bleeding. Thus, the influence of estrogen on TL and female longevity is still controversial.

There is *in vivo* and *in vitro* evidence suggesting that estrogens are able to induce TA and hTERT expression in the endometrium (Tanaka *et al*., 1998; Kyo *et al*., 1999; Vidal *et al*., 2002). In contrast, postmenopausal endometrium and endometrium treated with anti-estrogen drugs exhibited decreased TA (Tanaka *et al*., 1998). Furthermore, long term treatment with clinically relevant doses of conjugated E2 increased TERC expression preferentially in endometrial glands of ovariectomized female cynomolgus macaques (Macaca fascicularis) (Vidal *et al*., 2002). Increased TERC levels also correlate with higher proliferation and progesterone receptor expression in the endometrium of treated animals (Vidal *et al*., 2002). Both of these parameters are known to be regulated by estrogen in the endometrium (Hapangama *et al*., 2015). In the ER positive, hormone responsive endometrial epithelial adenocarcinoma cell line (Ishikawa cells), E2 induced TA and hTERT mRNA levels *via* a MAPK dependent pathway in an ERα dependent fashion (Zhou *et al*., 2006). In contrast, isolated primary epithelial cells (Tanaka *et al*., 1998) or intact endometrium in short-term explant culture did not show a significant response to E2 on TA (Valentijn *et al*., 2015). Conversely, co-cultured primary endometrial epithelial and stromal cells responded to E2 or a mitogenic fibroblast growth factor stimulus, suggesting that the E2 effect on telomerase induction may be enhanced or mediated by stroma and/or the duration of E2 treatment (Oshita *et al*., 2004).

#### Progesterone

Although progesterone has diverse effects on hTERT mRNA expression in progesterone receptor (PR) expressing breast and EC cell lines, the mechanisms by which hTERT expression is regulated by progesterone appear to be complex. The hTERT promoter lacks a canonical progesterone-responsive element (Wang *et al*., 2000), therefore classical PR mediated direct effects are less likely. The role of recently described progesterone receptor membrane components 1 and 2 on telomerase regulation has not yet been demonstrated (Bunch *et al*., 2014). In a breast cancer cell line, synthetic progestogen, medroxyprogesterone acetate inhibited hTERT mRNA transcription even in the presence of estrogen (Wang *et al*., 2000; Lebeau *et al*., 2002) and arrested cells in the late G1 phase (Lebeau *et al*., 2002) with the induction of p21 (Lange *et al*., 1999; Wang *et al*., 2000). There is also evidence for cell cycle-dependent regulation of telomere synthesis and telomerase gene expression in healthy hormone responsive human cells by progesterone (Wang *et al*., 2000; Tomlinson *et al*., 2006).

Since endometrial TA, hTERT mRNA/protein and hTERC levels reach their nadir during the progesterone-dominant mid-secretory phase, progesterone is thought to negatively regulate endometrial telomerase (Williams *et al*., 2001; Hapangama *et al*., 2008; Hapangama *et al*., 2009; Valentijn *et al*., 2015). The shortest endometrial TLs were also measured in the mid-secretory phase, indicating a telomere lengthening/maintenance function for endometrial TA (Valentijn *et al*., 2015). Exogenous progestogen administration is known to inhibit endometrial epithelial proliferation (Kurita *et al*., 1998; Shimizu *et al*., 2010) and we have recently shown this progestogen-induced decreased endometrial cell proliferation to be associated with a significant decrease in TA (Valentijn *et al*., 2015). Interestingly, the inhibition of TA in the progesterone-dominant secretory phase is associated with an induction of endometrial p21 and corresponds to a non-DNA damage induced cell cycle arrest function of p21 (Toki *et al*., 1998; Yoshimura *et al*., 2007). We can speculate that progesterone induced telomerase suppression might result in short endometrial epithelial telomeres (Hapangama *et al*., 2009; Valentijn *et al*., 2015) and perhaps influence changes in the endometrial epithelial cell cycle *via* p21 induction (Aix *et al*., 2016). Taken together, the hTERT gene may be a target of progesterone and the well-established progesterone induced down-regulation of the endometrial cell cycle may involve telomerase.

#### Androgens

Androgens such as dihydrotestosterone (DHT) induced TA at the G1 phase of the cell cycle in the androgen sensitive prostate cancer cell line LnCAP (Thelen *et al*., 2004). However, there was no modulation of TA by androgens in either androgen insensitive prostate cancer cell lines (TSU-Pr1, DU145) or in normal human prostate cells (Soda *et al*., 2000). In a recent study in men, serum DHT and E2 levels were shown to correlate with TL in PMBC, suggesting that both hormones may have a synergistic influence on TA (Yeap *et al*., 2016). However, caution should be taken when interpreting this observation as the authors have not demonstrated a direct regulatory effect. Oral treatment with Danazol (a synthetic steroid with weak androgenic properties) for 2 years resulted in universal leucocyte telomere elongation in both male and female patients with diseases such as bone marrow failure, liver cirrhosis and pulmonary fibrosis known to involve telomeres (Townsley *et al*., 2016). The intra-cellular metabolism of testosterone to estrogens is well described (Sasano *et al*., 2008). Androgens appear to regulate telomerase expression and activity mainly by aromatization of testosterone to estrogens through ERα in normal peripheral blood lymphocytes and human bone marrow-derived CD34(+) cells *in vitro* (Calado *et al*., 2009). Therefore, it is difficult to clearly ascertain if the observed effects of androgenic compounds were related to the direct effects on the androgens receptor (AR) or indirectly mediated *via* ER.

Endometrium expresses AR yet the direct specific effects of androgens in normal endometrium are only beginning to be understood. There is no published work yet examining the effects of androgens in endometrial telomerase regulation.

#### Other hormones relevant for the endometrium

Melatonin appears to regulate hTERT and hTERC expression in MCF-7 cells (Leon-Blanco *et al*., 2004) while dexamethasone reduced TA through the inhibition of TERT expression before induction of apoptosis (Akiyama *et al*., 2002). In contrast, hydrocortisone did not affect TA in human leucocytes (Calado *et al*., 2009). Therefore, the evidence for other non-ovarian steroidal hormones having a potential regulatory function of endometrial telomerase is limited and they will not be further discussed in this review.

### Endometrial stem cells and telomerase

The involvement of SPCs in the endometrial regenerative process has been suggested for a long time (Prianishnikov 1978). After menstrual shedding, a new functionalis layer is thought to regenerate from the remaining SPC rich basalis (Valentijn *et al*., 2013; Hapangama *et al*., 2015) and SPCs in many other tissues have the potential to activate telomerase (Hiyama and Hiyama 2007). Interestingly, the available evidence for the differences in TA between the endometrial basalis and the functionalis is controversial. A study in which different endometrial layers were crudely isolated by scraping (using a curette or a scalpel) suggested that TA is lower in the basalis (Bonatz *et al*., 1998); whereas isolated basalis epithelial cells, identified by expression of the surface marker SSEA1 from primary endometrial epithelial cells in short-term culture, showed higher TA than functionalis epithelial cells (Valentijn *et al*., 2013). Our study examined only sorted endometrial epithelial cells and telomerase expression and TA were limited mainly to the epithelial cells (Tanaka *et al*., 1998; Yokoyama *et al*., 1998; Valentijn *et al*., 2015). Therefore both studies should have demonstrated similar results. The reasons for this contradictory observation could be due to the fact that epithelial SPC cells are likely to be activated during isolation and cultivation in the latter study, which removed the epithelial SPC cells from its niche, a process known to induce telomerase (Engelhardt *et al*., 1997). Furthermore, the presumed basalis tissue obtained by scraping the myometrium in the former study might have contained a higher proportion of myometrial tissue with low TA levels.

The available evidence suggests that the endometrium contains multiple progenitor cell populations. Cells with some stem cell properties have been isolated from the human endometrium expressing phenotypical markers of epithelial, stromal, leucocyte and vascular origin (Chan *et al*., 2004; Masuda *et al*., 2010; Cervello *et al*., 2011). Freshly isolated undifferentiated side population cells containing all these primitive cell types from human endometrium also expressed TA (Cervello *et al*., 2011). The presence of TA in the most widely characterized and studied endometrial SPC cell subtype, the endometrial stromal (mesenchymal) SPCs (Gargett *et al*., 2016), is yet to be fully described. Human MSCs (hMSC) from other locations are known to have negative or very low TA (Zimmermann *et al*., 2003; Tichon *et al*., 2009; Ogura *et al*., 2014). However, there is a report suggesting that early passages of endometrial stromal SPCs isolated on the basis of their expression of the putative MSC marker CD146 express hTERT protein and mRNA but the authors did not measure TA (Yang *et al*., 2011). Furthermore, isolated primary endometrial stromal cells had low but measurable TA in our recent study (Valentijn *et al*., 2015). Our unpublished data also suggest that TA in isolated primary stromal cells positively correlated with mean TLs, suggesting that telomerase expression may have a telomere lengthening function in these cells (Fig. 3). It will be interesting in the future to confirm these preliminary findings and examine the functional relevance of TA to TL in the different endometrial SPC subtypes. However, the low amount of TA in stromal cells and problems with the specificity of the currently available anti-hTERT antibodies that are suitable for IHC/IF make this task difficult. Interestingly, in a recent study, there were rare telomerase expressing cells in murine endometrial stroma that may represent SPC cells (Deane *et al*., 2016). However, due to the fact that telomerase is expressed constitutively in many mouse tissues unlike in humans, it is difficult to evaluate the significance/relevance of this finding in the context of the human endometrium.

The only characterized human endometrial epithelial cell subpopulation (cells that express surface marker SSEA-1, nuclear SOX9 and nuclear β-catenin) that exhibits progenitor properties *in vitro*, also showed high TA and longer TL compared with their more differentiated epithelial cell counterparts (Valentijn *et al*., 2013; Gargett *et al*., 2016). Importantly, these cells with high TA were able to produce endometrial gland like structures in 3D *in vitro* culture and when confronted with a 2D environment they were able to produce a monolayer, functionally akin to the re-epithelialization of the denuded endometrial surface after shedding of the functionalis (Valentijn *et al*., 2015). Finally, in a study employing immunofluorescence microscopy, the potential stem cell marker Mushashi1 also co-localized with the telomerase protein hTERT in the endometrial epithelium (Gotte *et al*., 2008). However, Mushashi1 expressing cells have not been shown yet to have stem cell characteristics in functional studies.

Taken together, the above data suggests that endometrial SPC cells (basalis SSEA1+ epithelial cells and possibly stromal SPCs) have TA (Valentijn *et al*., 2015). The exact function of telomerase in the epithelial and in stromal SPCs is not fully understood yet. Similar to the intestine, epidermis and other epithelial tissues and organs, endometrium may also have multiple, heterogeneous epithelial stem cell populations with or without a functional hierarchy (Schepers *et al*., 2011; Goodell *et al*., 2015; Pirvulet 2015) and they may have corresponding differential telomerase activation states. Active, more differentiated progenitor cells involved in normal physiological regeneration might have higher TA, while the true, dormant/quiescent stem cell population may not express any or very low levels of TA until they are activated. The quiescent stem cell population may have low TA during normal physiological regeneration of endometrium and only show high TA if challenged by extensive tissue disruption or when progenitors are compromised, such as after iatrogenic endometrial ablation (Hiyama and Hiyama 2007; Biswas *et al*., 2015). Further studies on telomere biology and telomerase function in the endometrial stem cell population are required in order to elucidate altered pathways relevant to endometrial proliferative diseases. Since stem cells are hypothesized to harbour defects specific to chronic endometrial pathologies (Figueira *et al*., 2011; Gargett *et al*., 2014; Sourial *et al*., 2014), treatment strategies directed towards them may prove to be curative.

### Role of telomerase in pathological conditions of the endometrium

#### Endometriosis

Endometriosis is a common chronic inflammatory disease, defined by the existence of endometrial like stroma and epithelial tissue in ectopic sites, outside the uterine cavity. Since endometrial tissue is intensely responsive to ovarian hormones, the main stimulus for the growth of ectopic endometriotic lesions is estrogen (Sourial *et al*., 2014; Hapangama *et al*., 2015; Hapangama and Bulmer 2016; Kamal *et al*., 2016) and progesterone resistance has been proposed as a fundamental feature of ectopic endometriotic lesions (Bulun *et al*., 2006). The endometrium of women with endometriosis has been shown to be different to that of healthy fertile women (Hapangama *et al*., 2012; Mathew *et al*., 2016).

**Figure 4:**
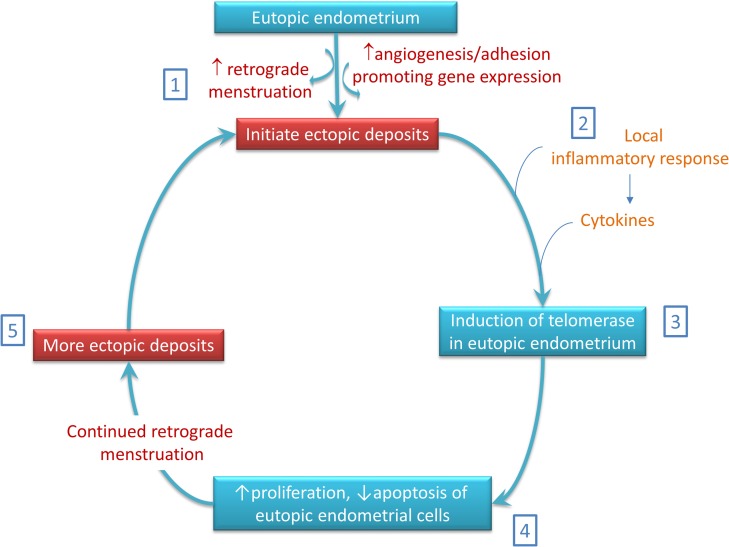
Telomerase is suggested to play a key role in our proposed model for the pathogenesis of endometriosis: (1) Ectopic endometriotic deposits are initiated by an increase in retrograde menstruation or an increased activity in genes that promote angiogenesis and adhesion. (2) The ectopic endometriotic deposits induce a local inflammatory response and secrete various cytokines. (3) The cytokines (or other substances) act on the eutopic endometrium to induce the pro-proliferative markers. (4) The induced eutopic endometrial cells express telomerase and adopt the pro-proliferative, apoptosis-resistant phenotype, which has a survival advantage in the peritoneal cavity. (5) Finally, retrograde menstruation of induced eutopic endometrium with the pro-proliferative phenotype together with other genes that also promote cell survival gives rise to further endometriotic deposits and maintains the disease (adapted from Hapangama *et al*., 2010).

There is a large body of evidence demonstrating that high TA, expression of hTERT and protein levels associated with longer mean endometrial TLs are features of the eutopic secretory endometrium of women with endometriosis (Kim *et al*., 2007; Hapangama *et al*., 2008; Hapangama *et al*., 2009; Hapangama *et al*., 2010; Valentijn *et al*., 2013; Mafra *et al*., 2014; Valentijn *et al*., 2015). These changes have been proposed to contribute to the functional endometrial abnormalities that result in the clinical manifestation of subfertility and propagate ectopic lesions. Considering the known pro-survival function of telomerase, the high TA in late-secretory endometrium of women with endometriosis (Hapangama *et al*., 2008) might be responsible for the survival of cells that are shed into the peritoneal cavity during retrograde menstruation (Hapangama *et al*., 2010). The preferential survival of these cells and their enhanced replicative capacity due to high TA could facilitate implantation and/or establishment of ectopic lesions (Hapangama *et al*., 2010; Valentijn *et al*., 2015) (Fig. 4). This corresponds well with the finding of high TA and hTERT mRNA/protein levels in active peritoneal ectopic endometriotic lesions. In addition, ectopic epithelial cells display longer relative TL compared with eutopic epithelial cells from the same patient (Hapangama *et al*., 2008; Valentijn *et al*., 2015). This observation seems to be in agreement with the progesterone resistance described in the pathogenesis of endometriosis (Bulun *et al*., 2006; Sourial *et al*., 2014), where the development of ectopic endometriotic lesions may increase TA due to the failure of endogenous progesterone to inhibit telomerase at the ectopic site. We have already shown that dysregulation of telomerase is an important early change in endometriotic cells since high TA was required for the early establishment of ectopic lesions in a baboon model of induced endometriosis (Hapangama *et al*., 2010; Afshar *et al*., 2013) (Fig. 4).

Furthermore, in the baboon model, establishment of ectopic lesions was associated with induction of high TA and TERT expression in the eutopic endometrium (Hapangama *et al*., 2010). Interestingly, the initial induction of endometriosis was associated with activation of epidermal growth factor (EGF) signalling in the eutopic endometrium of the baboon model (Afshar *et al*., 2013) and EGF signalling was associated with up-regulation of TA in normal ovarian surface epithelial cells (Bermudez *et al*., 2008). A similar scenario might be happening in the eutopic endometrium in the baboon model. Eutopic endometrial cells with high TA can subsequently initiate more ectopic lesions after retrograde menstruation contributing to a self-propagation cycle of the disease (Hapangama *et al*., 2010) (Fig. 4). Ovarian endometriotic epithelial cells were successfully immortalized by combinatorial transfection of human cyclin D1, cdk4 and hTERT genes, whereas the introduction of hTERT alone, or together with cdk4, was insufficient for immortalization of these cells (Bono *et al*., 2012). Therefore, telomerase alone may not be sufficient for the apparent survival advantage displayed by the endometriotic cells (Fig. 4).

SPCs are suggested to play a key role in the pathogenesis of endometriosis (Figueira *et al*., 2011; Gargett *et al*., 2014; Sourial *et al*., 2014). Intriguingly, the epithelial cells of ectopic lesions show phenotypic similarities with SSEA1 expressing basalis epithelial cells (Valentijn *et al*., 2013; Valentijn *et al*., 2015). Recent data also suggests that the tumour suppressor protein ARID1A might negatively regulate hTERT transcription and TA. Induction of ARID1A repressed transcription of hTERT *via* binding to a regulatory element on the hTERT promoter, and promoted a repressive histone mode *via* occupying SIN3A and H3K9me3 (Rahmanto *et al*., 2016). ARID1A is a member of the SWI/SNF chromatin remodelling complex, and is reported to be frequently mutated in two epithelial ovarian carcinoma subtypes: ovarian clear cell carcinomas and endometrioid ovarian carcinomas (Samartzis *et al*., 2013; Grandi *et al*., 2015). These cancers have been molecularly and epidemiologically linked to endometriosis with approximately 20% of benign ovarian endometriosis lesions having a loss of BAF250a (encoded by ARID1A) expression (Xiao *et al*., 2012). Therefore, it is conceivable that hTERT expression may be potentially involved in carcinogenesis associated with the loss of ARID1A. However, the seemingly vigorous endometrial regulation of telomerase *via* ovarian hormones and the fact that TA levels are high and constitutively expressed in proliferating endometrial epithelial cells may be responsible for the apparently rare incidence of such transformation (Zafrakas *et al*., 2014; Ness *et al*., 2015). TL in peripheral lymphocytes of women with endometriosis compared to healthy controls did not differ in our studies (Hapangama *et al*., 2008; Hapangama *et al*., 2009) while others have reported longer telomeres in PBMCs from women with endometriosis (Dracxler *et al*., 2014). The reason for this observed difference could be attributed to the influence of different demographic features known to affect PMBC TL such as age, BMI and ethnicity. These factors were not accounted for and significantly differ between the two patient groups in the latter study and may account for the different TLs observed rather than endometriosis. Our studies controlled for these demographical parameters as well as the menstrual cycle phase and did not show an endometriosis associated significant difference in PMBC TLs (Hapangama *et al*., 2008; Hapangama *et al*., 2009).

Endometriosis shares some of the typical features of increased synthesis of pro-inflammatory cytokines and the imbalance between pro-inflammatory and anti-inflammatory cytokines with other chronic inflammatory diseases (Fig. 4, Souriel *et al*.2014). Interestingly, telomere attrition and decreased TA have been associated with many chronic inflammatory diseases (Zhang *et al*., 2016). Although opposite changes in endometrial telomere regulation and involvement of high TA in endometriosis have been confirmed by various studies to date (Kim *et al*., 2007; Hapangama *et al*., 2008, 2009, 2010; Valentijn *et al*., 2013; Mafra *et al*., 2014; Valentijn *et al*., 2015), this knowledge is yet to be translated into a therapeutic solution. Since endometriosis is postulated to be a progesterone resistant condition (Bulun *et al*., 2006) and since telomerase inhibition is a downstream effector of progesterone (Valentijn *et al*., 2015), the option of telomerase inhibition must be further explored as an attractive, non-hormonal treatment for endometriosis.

#### Endometrial polyps

Endometrial polyps are defined as abnormal outgrowth of hypertrophic endometrial tissue consisting of a monoclonal overgrowth of endometrial stromal cells with inclusion of a non-neoplastic glandular component (Hapangama and Bulmer, 2016). Endometrial stimulation by estrogen is postulated as the main driving force for endometrial polyp formation and this is supported by the observation that the use of tamoxifen, which acts as an ER agonist on the endometrium, increases the risk of developing endometrial polyps. Lower levels of hTERT protein in endometrial polyps have been reported compared with normal endometrium in the proliferative phase (Hu and Yuan, 2011). CD146 expressing mesenchymal SPCs derived from endometrial polyps did not express any hTERT (Ding *et al*., 2011). Jointly these studies suggest that benign endometrial polyps with increased stromal growth have low telomerase and that TA is less likely to play an essential role in them. However, telomerase biology in polyps with epithelial hyperplasia/atypia remains to be explored in future studies.

#### Reproductive failure

Embryo implantation occurs during the window of implantation in the mid-secretory phase. The mid-secretory phase is defined by the dominant action of progesterone with maximum cell differentiation in an environment where endometrial glandular proliferation indices are at their nadir. This period is also associated with the lowest endometrial TA and the shortest mean TL (Williams *et al*., 2001; Valentijn *et al*., 2015) suggesting a requirement of low endometrial TA for the establishment of an early pregnancy. This suppression of TA in the endometrium of fertile women has been proposed as a necessary process in order to allow endometrial cells to undergo differentiation with cellular apoptosis/senescence required to make space for the invading embryo (Williams *et al*., 2001; Hapangama *et al*., 2008; Hapangama *et al*., 2009; Hapangama *et al*., 2010). It is therefore not surprising that significantly high telomerase expression and TA was observed in endometrial tissue of women with recurrent reproductive failure (Hapangama *et al*., 2008; Long *et al*., 2016). The mid-secretory endometria of women with recurrent miscarriages, recurrent embryo loss and recurrent implantation failure all showed high endometrial TA (Hapangama *et al*., 2008; Long *et al*., 2016) and a trend for longer mean endometrial TLs in endometrial epithelium (Hapangama *et al*., 2008). However, this preliminary evidence needs to be confirmed in independent studies. It is plausible that persistent proliferation and high TA of endometrial cells may interfere with the embryo implantation and trophoblastic invasion, all of which are known to be involved in the establishment of early pregnancy. The question of why TA is down-regulated in the endometrium of successful pregnancies but not so in unsuccessful cases implies a difference in telomerase regulation. So far, the underlying mechanisms for the differential (dys)regulation are not understood. In a further case controlled study, infertile women with deep infiltrating endometriosis also had high endometrial telomerase expression further suggesting a detrimental effect of high levels of TA on conception/embryo implantation (Mafra *et al*., 2014). Progesterone is commonly employed to treat women with a variety of reproductive failures, from infertility, to luteal phase defects to recurrent miscarriages, yet the available evidence on the effectiveness of this therapy is inconclusive (Coomarasamy *et al*., 2016). Most of these conditions are multifactorial and the lesions from women included in clinical trials therefore are heterogeneous. This prevents elucidation of the true effectiveness of progesterone treatment in subgroups of women with an apparently similar clinical manifestation. Further examination of downstream effectors of progesterone treatment, such as telomerase, may enable the stratification of women in the future in order to identify those who may benefit from the administration of the hormone.

#### Polycystic Ovarian Syndrome

PCOS is a common gynaecological condition defined by the clinical manifestations of hormonal aberrations of hyperandrogenism and insulin resistance (Clark *et al*., 2014; Lizneva *et al*., 2016). It is often associated with anovulation and a subsequently increased risk of EH with risk of progression to EC (Hapangama *et al*., 2015; Hapangama and Bulmer, 2016; Kamal *et al*., 2016). A genome-wide association study in Korean women with PCOS has identified susceptibility loci for polycystic ovarian syndrome (PCOS) (Lee *et al*., 2015). The authors reported the strongest signal to be located upstream of KH domain containing, RNA binding, signal transduction associated 3 (KHDRBS3). KHDRBS3 was found to regulate TA in colon cancer cells (Zhang *et al*., 2006). With this evidence, the authors concluded that telomerase may be an important driving force in developing PCOS and related phenotypes (Lee *et al*., 2015). Considering the suggested role that unopposed estrogen and excessive androgens have on TA (Boggess *et al*., 2006; Nourbakhsh *et al*., 2010) and cellular proliferation in various carcinoma cells including EC cells (Chao *et al*., 2013; Dumesic and Lobo, 2013; Plaza-Parrochia *et al*., 2014; Hapangama and Bulmer, 2016; Kamal *et al*., 2016), studies examining the involvement of telomerase in the endometrium of women with PCOS may unravel novel avenues for therapeutic manipulation.

#### Malignant conditions of the endometrium

Constitutively high levels of hTERT expression and TA have been identified in over 90% of human cancers including hepatocellular carcinoma, colorectal cancer and EC (Lehner *et al*., 2002; Saini *et al*., 2009; Bertorelle *et al*., 2014). High levels of TA in tumour cells contribute to increased cell proliferation, cellular immortality, carcinogenesis and cancer progression, while the activity of telomerase and hTERT expression are usually suppressed in most human somatic tissues (Cong *et al*., 1999). The involvement of telomerase in cellular immortality is further highlighted by the fact that most cell lines, including the first ever ‘immortal cell line’, cervical HeLa cells express very high levels of TA (Pandita *et al*., 1997). The involvement and activation of telomerase and telomere maintenance during tumorigenesis has been intensely studied over the years. In HeLa cells, as in many cervical cancers, the mechanism of telomerase activation is regulated by specific proteins from human papillomavirus Types 16 and 18 (Kyo *et al*., 1996; Sakamoto *et al*., 2000; James *et al*., 2006). Other cancers that do not involve viral infection in the pathogenesis also demonstrate high TA but the exact mechanism of their telomerase activation is not fully understood. Telomerase suppressing mechanisms that are downstream of hTERT transcription and mRNA splicing are present in rapidly proliferating embryonic tissue (Ulaner *et al*., 2000), but these are lost during neoplastic transformation (Ulaner *et al*., 2000). Therefore, the tight physiological mechanisms of telomerase regulation do not exist in cancer cells where TA levels remain constitutively high.

There are reports of shorter or longer TLs in different cancer cells compared with their benign counterparts. Short TLs in cancer cells may result from excessive cell proliferation prior to telomere stabilization (with TA or an alternative telomere maintenance mechanism (ALT)). Telomere attrition can result in genomic instability which can subsequently initiate carcinogenesis. Absolute TL is therefore not relevant for cancer cells as long as telomeres are sufficiently maintained in a capped state in order to supply the cells with an indefinite proliferation capacity. There are at least four activating mutations reported in TERT, POT1, TPP1 and TERF2IP (RAP1) genes of the telomerase and telomere complexes which can result in longer TLs while several other telomere and telomerase associated gene mutations (including repressor mutations in POT1 and activating mutations of TRF1/2) result in short TL (reviewed in Mengual Gomez *et al*., 2016). Since either lengthening or shortening of telomeres can result in abnormal cell proliferation or genomic instability, they can be implicated in carcinogenesis (reviewed in Holysz *et al*., 2013; Mengual Gomez *et al*., 2016; Shay, 2016). In this review, we examine the available evidence for specific aberrations in telomeres/telomerase in the premalignant and malignant conditions of the endometrium.

#### Endometrial hyperplasia

EH is characterized by irregular proliferation of endometrial glands that may precede or co-exist with endometrial carcinoma (Hapangama and Bulmer 2016; Kamal *et al*., 2016). Hyperplastic glands show extremely high proliferative indices in comparison to either stromal cells or normal glands during the proliferative phase (Dallenbach-Hellweg and Dallenbach, 2010). EH is almost always due to exposure to high estrogen levels and typically accompanied by a chronic insufficiency of progesterone. Classical causes therefore include corpus luteum insufficiency/anovulatory cycles, PCOS, obesity with metabolic syndrome (extra-ovarian aromatization of androgens) and inappropriate postmenopausal hormone therapy (tamoxifen, insufficient dosage of progestagens) (Hapangama and Bulmer 2016; Kamal *et al*., 2016).

High TA levels have been detected in EH, including the simple, complex and complex with atypia subtypes (Shroyer *et al*., 1997). It was also suggested that TA could be a useful diagnostic tool to screen postmenopausal women with endometrial premalignant and malignant lesions (Maida *et al*., 2002). However, there is a considerable proportion of EH samples included in these studies that lack TA (Shroyer *et al*., 1997) and the absence of detectable TA did not have a specific negative predictive value (Shroyer *et al*., 1997). Considering the method used to sample the endometrium (for example, an outpatient endometrial biopsy typically samples approximately 4% of the uterine cavity), it is unlikely that a small area of EH is reliably sampled and detected with this approach. Furthermore, since TA is a feature of normal proliferating endometrial cells, including simple hyperplasia, the level of TA is unlikely to be a sufficient discriminator to detect malignant or premalignant conditions of the human endometrium.

#### Endometrial cancer

Most previous research on endometrial telomerase had been focussed on EC that is associated with high TA. EC is the commonest gynaecological malignancy and is an estrogen driven disease (Kamal *et al*., 2016). The risk factors for EC include advanced age, obesity and exposure to unopposed estrogen (or progesterone deficiency). It is of interest that despite the high estrogenic milieu associated with the premenopausal period where most vigorous proliferative and regenerative activity takes place in the endometrium, carcinogenesis commonly occurs in the relatively quiescent, hypo-estrogenic postmenopausal period (Hapangama and Bulmer 2016; Kamal *et al*., 2016).

Very low but detectable TA levels are reported in the postmenopausal endometrium (Tanaka *et al*., 1998). Estrogen induces telomerase in a dose dependent manner (Kyo *et al*., 1999). Various risk factors (such as obesity) for EC will marginally increase the weak estrogen (estrone) levels in the postmenopausal endometrium (reviewed in Kamal *et al*., 2016). The intermittent and low levels of estrogen associated with these conditions (Kamal *et al*., 2016) may be sufficient for inducing low TA levels to initiate epithelial proliferation in the postmenopausal endometrial cells. Furthermore, both obesity and ageing are chronic inflammatory conditions that are associated with oxidative stress, thus accelerating telomere shortening in proliferating cells (von Zglinicki *et al*., 1995; von Zglinicki 2000; Kamal *et al*., 2016). Therefore the most likely mechanism to explain EC associated telomere aberrations is the insufficient TA/short-TL/damaged telomeres theory. We propose that the hypo-estrogenic hormonal maelstrom in high risk postmenopausal endometrium may be associated with a potentially deficient amount of TA that is insufficient to maintain the short endometrial TLs and their integrity during cell division. Therefore, the ongoing proliferation in postmenopausal epithelial cells with short TLs may render them vulnerable to subsequent genetic instability and carcinogenic transformation similar to various other cancer types (Greider and Blackburn 1996; Bertorelle *et al*., 2014; Meena *et al*., 2015). In line with the above hypothesis, short TLs have been reported in most sporadic ECs.

Lynch syndrome is an autosomal dominant condition characterized by germ line mutation in DNA mismatch repair genes resulting in increased risk of developing a variety of cancers including EC. Long telomeres are associated with familial cancer syndromes such as familial melanoma (Akbay *et al*., 2008; Horn *et al*., 2013). However, short TLs and hTERT gene polymorphisms predict initiation of EC in Lynch patients (Segui *et al*., 2013).

High TA, hTERT and hTERC expression as well as high hTERT protein levels have been described by many groups in ECs (Kyo *et al*., 1996; Saito *et al*., 1997; Ebina *et al*., 1999; Maida *et al*., 2002). Some preliminary data in a very small patient population also suggests that hTERT mRNA in PBMCs can be used to diagnose early micro-metastases of EC (Liang *et al*., 2016). This observation needs further validation in an appropriately powered study. Frequent PTEN mutations and P53 loss known to occur in ECs could be associated with telomerase up-regulation but the exact mechanism through which these different events interact is not yet clear (Zhou *et al*., 2006; Akbay *et al*., 2013). Further recent work also showed hTERT to be involved in EMT, cell motility and metabolism, which are processes associated with cancer metastasis (reviewed in Saretzki, 2014). In a mouse model, simultaneous deletion of p53 and POT1 resulted in precursor lesions of endometrial epithelium and induced ECs with non-endometrioid, Type II phenotype, suggesting that telomeric instability has a critical role to play in Type II ECs (Akbay *et al*., 2013). TERT promoter mutations seem to be rare in ECs (Wu *et al*., 2014), except for the clear cell (Type II EC) subtype (Huang *et al*., 2015). Activating TERT promotor mutations are a feature of cancers derived from tissues with low relative cellular turnover such as the brain, therefore such mutations are not expected in the highly regenerative endometrial epithelium (Killela *et al*., 2013; Shay 2016).

There are limited reports suggesting that the ALT pathway may be active in some endometrial carcino-sarcomas or uterine sarcomas (Lee *et al*., 2012), but its prevalence in endometrioid ECs appears to be low (Heaphy *et al*., 2011). Further studies are required to dissect out the exact function high TA plays in ECs and how telomerase regulation in the postmenopausal endometrium may induce carcinogenesis, particularly, in the context where a high risk hormonal environment is present. In the light of many reported alterations in the components of the telomere complex, telomerase holoenzyme and their associated proteins in cancer (Greider and Blackburn, 1996; Shay, 2016), in addition to the complex interactions of these changes with numerous tumour suppressor proteins and oncogenes, more detailed studies are required to fully understand the role of alterations in the telomere maintenance pathways particular to all subtypes of EC.

## Future directions and wider implications of interventions into telomerase biology

hTERT has been reported to be up-regulated by various pharmaceutical and metabolic agents such as angiotensin-converting enzyme inhibitors (Donnini *et al*., 2010), essential fatty acids (Das 2008), beta blockers (Wang *et al*., 2011) and calcium channel blockers (Hayashi *et al*., 2014) in a variety of tissue types. Telomerase inhibition has been reported as an off-target effect of various chemotherapeutic agents, however this it could be a secondary effect to apoptosis or senescence induction (Saretzki, 2010). Imetelstat is the only clinically applicable synthetic telomerase inhibitor, which is a lipid based conjugate of the oligonucleotide GRN163 that binds with high affinity to the hTERC component of telomerase (Roth *et al*., 2010; Salloum *et al*., 2016). Imetelstat had shown promising pre-clinical telomerase inhibitory activity across a wide range of cancer types (Roth *et al*., 2010). However, despite high expectations, all reported data on its clinical effectiveness in at least six different clinical cancer trials have been disappointing (Salloum *et al*., 2016), with treatment resulting in significant side effects such as myelo-suppression. Findings from a recent small clinical pilot study demonstrate a possible specific beneficial effect of Imetelstat in clinical outcomes in myelo-proliferative conditions that needs to be further confirmed in larger trials (Baerlocher *et al*., 2015). The side effect profile of this drug, however, precludes its use in benign endometrial conditions. The examination of different compounds with off-target inhibitory effects on TA and a more favourable side effect profile is required before clinical application in endometrial disease.

Our understanding of telomerase and telomere biology is rapidly expanding with many groups working towards developing this exciting field, as telomeres and telomerase pertain to a diverse range of cellular functions and processes. In addition, telomerase regulation and function in the human endometrium appears to be unique. The majority of treatments we have available at present for common benign and chronic diseases of the endometrium such as heavy menstrual bleeding, infertility, polyps, endometriosis and EH are directed towards terminally differentiated endometrial cells of the endometrial functionalis. The functionalis is shed each month with menstruation, thus requiring continuous therapy for each new functionalis layer that is regenerated. Most currently available therapeutic options therefore include ovarian or other hormonal analogues to manipulate the ovarian cycle resulting in considerable side effects. Since telomerase appears to be involved in almost all endometrial pathologies, it provides a distinctive route to formulate possible curative (involving stem cells), non-hormonal therapies to treat them. Furthermore, our current understanding of telomere and telomerase biology in ECs is far from comprehensive and the fact that high TA is present in normal proliferating endometrial cells makes it difficult to extrapolate the data from other cancers to ECs. TA in normal endometrium has strict physiological regulation, mainly by hormones. During tumourigenesis this regulation becomes dysfunctional, supplying cells with a constitutively high amount of TA, conferring a selective advantage and a high proliferative capacity. In addition, telomerase and telomere biology of the endometrial cells may be modified by the cellular interactions in a new environment (e.g. endometriotic lesions growing in the peritoneal cavity). A better understanding of these processes may facilitate formulating new interventions. Further studies in this field for both benign and malignant diseases of the endometrium should therefore focus on understanding the precise regulation mechanisms for telomerase which could potentially reveal novel targets for new treatment strategies based on telomerase in endometrial disease.

## Conclusion

We are only beginning to understand the central role of telomeres and telomerase in the biology of the endometrium. Since telomerase is pivotal to endometrial regeneration, further studies elucidating the involvement of telomere and telomerase associated proteins and their regulation in normal endometrial regeneration, and in endometrial pathologies, will help in developing novel treatment strategies that are not only non-hormonal but also potentially curative.
